# Single-molecule localisation microscopy (SMLM) is feasible in human and animal formalin fixed paraffin embedded (FFPE) tissues in medical renal disease

**DOI:** 10.1136/jcp-2024-209853

**Published:** 2025-01-13

**Authors:** Scarlet F Brockmoeller, Hayley Slaney, Alistair Curd, Aurora Bono, James H Felce, Deep Arora, Andrew Lewington, Andras G Miklosi, Philip Quirke

**Affiliations:** 1 Pathology & Data Analytics, Leeds Institute of Medical Research at St. James’s, School of Medicine, University of Leeds, Leeds, LS9 7TF, UK; 2 Department of Histopathology, Queen Elizabeth Hospital, Gateshead and Department of Precision and Molecular Pathology,Newcastle University, UK; 3 School of Molecular and Cellular Biology, Faculty of Biological Sciences, University of Leeds, LS2 9JT, UK; 4 ONI UK, Linacre House, Banbury Road, Oxford, OX2 8TA, UK; 5 Department of Histopathology, Bexley Wing, Level 5, St James's University Hospital, Leeds, LS9 7TF, UK; 6 Renal Department, St. James's University Hospital, Leeds, LS9 7TF, UK

**Keywords:** KIDNEY, Methods, Microscopy, Fluorescence, MOLECULAR BIOLOGY, NEPHROLOGY

## Abstract

**Aims:**

Establishment of a protocol for routine single-molecule localisation microscopy (SMLM) imaging on formalin fixed paraffin embedded (FFPE) tissue using medical renal disease including minimal change disease (MCD) and focal segmental glomerulosclerosis (FSGS).

**Methods:**

Protocol for normal and diseased renal FFPE tissue was developed to investigate the clinical diagnostic potential of SMLM. Antibody concentrations were determined for confocal microscopy and transferred to SMLM. Different fixatives and lengths of fixation were studied. To reduce autofluorescence, additional quenching and UV bleaching steps were compared. Optimal SMLM acquisition settings were established. SMLM data were imaged, digitally captured, stored, visually inspected and analysed quantitatively.

**Results:**

Protocol was established on normal renal FFPE tissue and then applied to clinical diseased tissue with single and multiple markers. Antibodies against key diagnostic proteins including podocin, nephrin, collagen, laminin, synaptopodin, CD31, IgG, IgM and IgA antibodies were established for MCD, FSGS and immune-mediated renal disease. We found important characteristic differences in the renal diseases listed above.

**Conclusions:**

We established a routine super-resolution microscopy protocol for clinical FFPE material on medical renal biopsies, which could visualise fluorescently labelled proteins in all glomeruli present with a precision of approximately 10–20 nm, with a turnaround under 48 hours. We visualised and quantitated specific protein distributions in different conditions. SMLM opens subcellular microscopy in FFPE to histopathologists on routine FFPE tissue, which can in the future be an adjunct and, in some aspects, a rapid superior alternative to electron microscopy.

**WHAT IS ALREADY KNOWN ON THIS TOPIC**

- Super-resolution fluorescence imaging, in particular single-molecule localisation microscopy (SMLM), can locate specific molecules in biological structures to within 10 nm. However, this technology is currently mainly used in research settings on cell culture material.
- SMLM has already been used in various cell culture applications to study a diverse range of targets and structures, with some applications also in biological tissue. However, we need to develop protocols for use on routine formalin fixed paraffin embedded (FFPE) tissue.

**WHAT THIS STUDY ADDS**

- We established a routine SMLM protocol for clinical FFPE material on medical renal biopsies, which could visualise specific proteins in all glomeruli present down to a precision of approximately 10 nm, with a turnaround under 48 hours.

**HOW THIS STUDY MIGHT AFFECT RESEARCH, PRACTICE OR POLICY**

- SMLM opens up subcellular microscopy in FFPE to histopathologists on routine FFPE tissue, which might in the future be an adjunct and, in some aspects, a rapid superior alternative to electron microscopy.

## Introduction

### Background

 Histopathology is based on the use of formalin fixed paraffin embedded (FFPE) tissue, which while practical for light and confocal microscopy, presents major challenges for ultrastructural imaging. This is due to the ultra-resolution imaging technique electron microscopy (EM), requiring specific methods for the fixation, embedding of tissue in resin, only viewing small areas of tissue and the expense of the equipment. The aim of this study was to achieve the potential of whole slide spatial resolution of 10–20 nm on routine retrospective clinical FFPE tissue, and hence open diagnostic nanoscale imaging to the pathology community.

Until recently, light microscopy techniques could not resolve details smaller than 250 nm due to the diffraction limit of light. A range of super-resolution techniques, including stimulated emission depletion microscopy, structured illumination microscopy and direct stochastic optical reconstruction microscopy, have now enabled the visualisation of cellular ultrastructure’s with 20–100 nm resolution,^1^,^4^ including in FFPE tissue samples.^5^ Super-resolution microscopy and detection of nanoscale changes have exciting potential for clinical diagnosis in a range of disease areas including medical renal,^6^ oncology, neurological, muscular and infectious disease. Bench-top microscopes with super-resolution imaging capabilities including single-molecule localisation microscopy (SMLM) have already been used in various cell culture applications to study a diverse range of targets and structures.^1 2^ In this study, we investigated its potential in routine archival clinical FFPE tissue and compared this with the gold standard EM.

Minimal change disease (MCD) and focal segmental glomerulosclerosis (FSGS) are two common forms of medical renal disease, where high-resolution imaging is helpful in establishing the diagnosis and excluding common differential diagnoses.^7^ Both are currently diagnosed on a kidney biopsy including routine histomorphology, immunohistochemistry, immunofluorescence and EM. Both diseases are characterised on EM by specific podocyte features, including foot process effacement 7–9^8^ and lack of electron dense deposits. Newer studies are indicating an immune-mediated pathology.^9^ EM requires special sample preparation and centralised facilities available only at a few centres in the UK. This is a costly and time-consuming test, with variable turnaround times of up to 2–3 weeks if facilities are not available locally.

Super-resolution microscopy, such as SMLM, may enable a more rapid, cheaper, decentralised platform test on FFPE tissue than EM. It can also examine the whole slide of a tissue section, including all the glomeruli present, in a renal biopsy. There is the option to quantify on a protein level the changes that occur at the nanoscale.^10^,^12^ All these factors together are promising for increasing diagnostic sensitivity and specificity, yielding benefits for patients and the National Health Service (NHS), also with the potential to aid in establishing and quantifying new markers for disease progression, relapse, early recurrence of disease and sensitivity to drug treatment.

## Methods

###  Tissue fixation

In routine diagnostic practice, standard guidelines are in place that are potentially limiting any variation in the fixation time, but in practice, tissue samples can be fixed for a variable time from 6 hours to 5 days or longer. Our ‘normal’ renal tissue was obtained at kidney donation retrieval, where they had been excluded from transplantation use and made available for research. Samples were fixed for 24 hours in formalin and then transferred to 70% ethanol, where they remained at room temperature until tissue processing.

The fixatives 1-6 were made up as follows^13^:

1. Neutral buffered formalin: 2 g sodium dihydrogen phosphate monohydrate, 3.25 g disodium hydrogen phosphate anhydrous and 450 mL deionised water.
2. Formol saline: 450 mL deionised water, 50 mL 37% formalin and 4.5 g sodium chloride.
3. Clarke’s fixative: 240 mL ethanol and 80 mL glacial ethanoic acid.
4. Carson’s fixative: 325 mL ethanol, 125 mL deionised water and 50 mol 37% formalin.
5. Bouin’s solution: 150 mL saturated picric acid solution, 50 mL 37% formalin and 10 mL glacial ethanoic acid.
6. 4% paraformaldehyde: this solution was prepared using an established in-house protocol and made fresh on the day of use. 1 M phosphate buffer was prepared by making up the following solutions: A: 7.05 g of disodium hydrogen phosphate dissolved in 500 mL deionised water and B: 6.9 g of sodium dihydrogen phosphate monohydrate dissolved in 500 mL deionised water. Solution A was added to Solution B until pH 7.3 was reached. 6 g of paraformaldehyde was dissolved in 150 mL of 1 M phosphate buffer by heating on a hotplate. Six drops of 0.1 M sodium hydroxide solution was added until the solution was clear, ensuring that the solution was kept below the boiling point.

In routine diagnostic practice, renal biopsy samples are immersed in neutral buffered formalin for 24 hours. Samples were taken from mouse kidneys and fixed with neutral buffered formalin for variable times, as well as placed in each fixative type for 24 hours. These samples were stained with a primary sheep IgG antibody against human nephrin (AF4269, R&D Systems) and a secondary donkey anti-sheep IgG antibody labelled with Alexa Fluor 647 (A-21559, Thermofisher) for the comparisons.

### Preparation of tissue and mounting of slides

1.5H coverslips (Deckgläser, VWR international, Radnor, USA) were prepared by serial washing at room temperature with acetone for 5 min, 100% ethanol for 5 min and deionised water for 5 min. The coverslips were then placed in a sonicating water bath for 30 min, rinsed in deionised water, wrapped in aluminium foil and left overnight in a drying oven set to 37°C.

To increase the adhesion of the tissue sections, the coverslips were coated with a 3-aminopropyltriethoxysilane (APES, Scientific Laboratory Supplies) solution. Coverslips were immersed in 4% APES solution for 5 min at room temperature. They were then washed in two sequential pots of acetone and deionised water for 1 min, wrapped in aluminium foil and left overnight in a drying oven set to 37°C.

### Immunofluorescence staining protocol for SMLM

FFPE mouse tissue and renal biopsies were sectioned at 3 µm thickness, mounted on the prepared APES-coated coverslips, and placed overnight in a drying oven at 37°C. Prior to staining, the tissue sections were baked on a hotplate at 70°C for 30 min to ensure adherence to the coverslips. The tissue sections were deparaffinised and rehydrated through a series of solutions: xylene (4×3 min), 100% ethanol (2×3 min), 95% ethanol (3 min), 70% ethanol (3 min), 50% ethanol (3 min) and finally rinsed in running water (5 min). Additional H&E sections were scanned and reviewed by a histopathologist.

Optimal antigen retrieval methods were determined for each individual antibody, through a process of optimisation, starting with the data sheets provided by supplier. All selected antibodies worked efficiently with heat induced epitope retrieval, and a pressure cooker was used, as standard. The antigen retrieval solution was dependent on antibody used (please see online supplemental table 1).

The coverslips were transferred to pots containing the appropriate antigen retrieval solution and placed inside the pressure cooker, alongside a pot containing phosphate buffered saline (PBS, pluriSelect, El Cajon, California, USA). The coverslips were pressure-cooked at 125°C for 5 min, then held at 90°C. They were then transferred to the pot of PBS for 30 s to equilibrate, rinsed under running water for 5 min, incubated with 0.1% Tween-20 (Sigma) for 5 min, and washed once with PBS .

Quenching with ammonium chloride resulted in the greatest reduction of autofluorescence and was used for all data collection. 50 mM NH_4_Cl (0.078 g of NH_4_Cl (Aventor, VWR) in 30 mL of PBS) was added to the sample and incubated at room temperature for 15 min, following antigen retrieval. The sample was washed with PBS for 5 min.

Permeabilisation was performed by incubating the samples with 0.4% Triton X-100 (Alfa Aesar, Heysham, UK) in PBS for 45 min, before washing in PBS, two times for 5 min. The samples were blocked using a solution of 3% bovine serum albumin (BSA) and 20% serum (goat or donkey) for 1 hour at room temperature. The serum type is dependent on the secondary antibody used (see online supplemental table 1). The BSA was gently removed, without washing, and the primary antibodies were applied at optimised concentrations and incubation conditions (see online supplemental table 1). After gently shaking off the primary antibody, the samples were washed with PBS, three times, for 5 min. An appropriate secondary antibody was applied to the sample and incubated in darkness for 1 hour at room temperature. After gently shaking off the secondary antibody, the sample was washed three times with PBS for 5 min. Samples were stored in fresh PBS at 4°C in the dark, until imaging. The imaging commenced within an hour, with all acquisitions captured on the day the staining protocol was completed.

### Primary and secondary antibodies

The antibodies used are listed in online supplemental tables 1 and 2. Primary antibody titrations were conducted to determine the most appropriate concentrations for SMLM imaging with either one or two antibodies in combination. These experiments helped to overcome fixation artefacts, light scattering and obtain good signal-to-noise imaging using SMLM on FFPE tissue.

### Antibody conjugation

10 µg of antibody was diluted in 100 mM carbonate buffer (Sigma-Aldrich, catalogue numberS5761) and 2 µL of the reactive dye (A20006, Thermofisher) dissolved in anhydrous dimethyl sulfoxide (DMSO) at 1 mg/mL concentration was added to the reaction mixture. The vial containing the reaction mixture was placed in a heating block for 30 min at 37°. The reaction was conducted under constant agitation and protected from light. After labelling, unreacted dye was removed by passing the reaction volume through three 40 kDa size exclusion columns (A57759, Thermofisher) washed with PBS (Thermofisher, catalogue number 10010023). After the cleanup, NaN_3_ (Sigma Aldrich, catalogue number 822335) was added to the conjugated antibodies at 0.02% concentration to prevent bacterial growth.

### Imaging of diseased medical renal samples (MCD, FSGS)

The optimised protocol was transferred into diseased medical renal tissue, including MCD and FSGS. FFPE blocks of FSGS and MCD cases were stained with H&E if possible and then scanned to allow comparison with the SMLM data. All glomeruli were reviewed on H&E and counted before imaging with SMLM imaging.

Further serial sections were imaged for podocyte integrity and cytoskeleton markers. The whole biopsy was imaged with SMLM including selected diseased glomeruli or all glomeruli present(10–30 glomeruli per case) within a single FFPE section. Where possible, the SMLM slide was subsequently stained with H&E to provide an accurate map of the imaged glomeruli. This was not always possible due to occasional tissue damage after imaging.

### SMLM imaging and analysis

Prior to imaging, a hydrophobic barrier pen was used to create a barrier around the sample. 100 µL of BCubed imaging buffer (ONI, Oxford, UK) was freshly made, as per manufacturer’s instructions, and applied to the sample. A second coverslip was gently laid over the section, sandwiching the buffer and tissue between the two coverslips.

Single molecule data acquisition (SMLM) was conducted on the Nanoimager S running NimOS V.1.19.4 (ONI, Oxford, UK). The images were acquired using a 100X 1.4 NA objective lens (Olympus, Japan) and a sCMOS camera (Hamamatsu Orca Flash 4.0 V3). Each acquisition was conducted in total internal reflection fluorescence illumination, with the exposure time set at 30 ms, with the temperature control enabled and set at 32°C.

The 640 nm laser was used to image the Alexa Fluor 647 dye, while the 561 nm laser was used to image the CF568 dye. All datasets contained 10 000 frames. Dual colour data were acquired sequentially with 640 nm, then 561 nm excitation lasers, with 5000 frames acquired per laser.

Drift correction, localisation filtering (to reduce background localisations) and some data analyses were performed in the cloud-based data analysis platform from the microscope manufacturer (CODI, ONI, Oxford, UK). Drift was corrected over all frames and included all localisations with photon count <30 000, SD of the point-spread function between 75 nm and 200 nm, estimated localisation precision (SD) <15 nm. Localisations were excluded where the same emission event lasted more than 15 frames.

Clustering used HDBSCAN^9^,^11^ with a minimum of 15 localisations per cluster and a minimum of 15 samples in neighbourhood calculations for the localisations. Cluster area (nm^2^) was used to quantify differences between healthy and pathological cases. Statistics and data visualisation were performed in GraphPad Prism V.10 for MacOS (GraphPad Software, Boston, Massachusetts, USA). Two-way anaysis of variance (ANOVA) followed by Tuckey’s multiple comparisons test was used to assess differences across samples in a pairwise manner.

### EM preparation of renal tissue samples and EM imaging

The EM report data and blocks from diseased cases (MCD, FSGS) were obtained through the Leeds Teaching Hospital Trust archive. Blocks were sent to the University Hospital of Leicester Trust, where the Specialist Scientific Lead for Electron Microscopy (Tracey de Haro), re-cut the resin blocks and obtained new EM images. The H&E slides of cases as well as EM images were reviewed and discussed.

## Results

### Impact of fixation solution and fixation time on quality of super-resolution imaging of tissue sections

**Figure 1:**
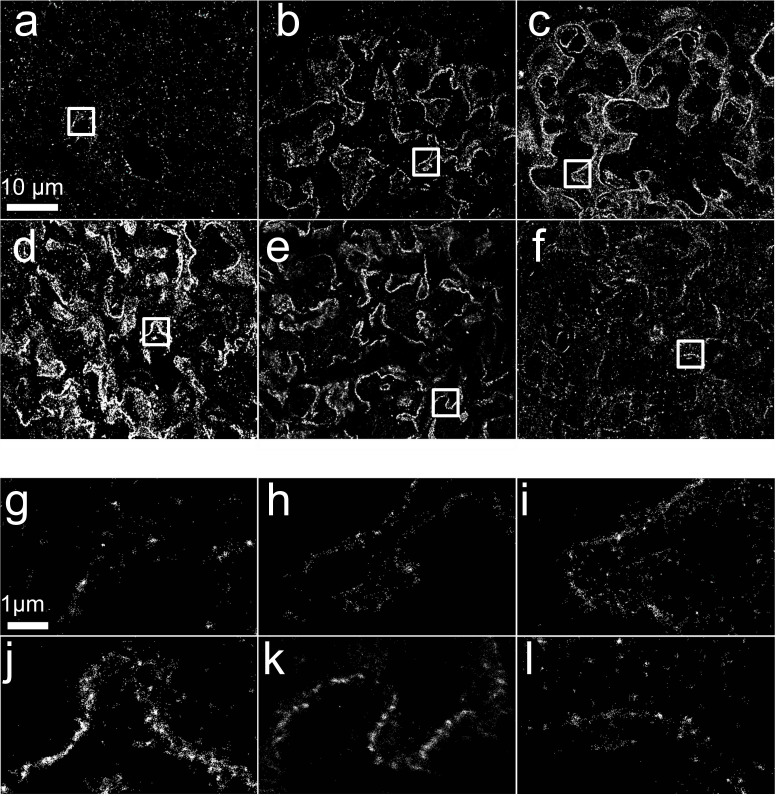
SMLM data from mouse kidney sections fixed with six different fixatives for 24 hours. (a, g) Bouin’s fixative, (b, h) Carson’s fixative, (c, i) Clarke’s fixative, (d, j) formol Saline, (e, k) neutral buffered formalin and (f, l) 4% paraformaldehyde. Boxed regions of (a)–(f) shown in (g)–(l). SMLM, single-molecule localisation microscopy.

We compared six different fixatives for mouse kidney: neutral buffered formalin, formol saline, Clarke’s fixative, Carson’s fixative, Bouin’s solution and 4% paraformaldehyde (figure 1). Resulting SMLM images of tissue sections labelled with nephrin, and 4', 6 - diamidino-2-phenylindole (DAPI) were of inferior quality for Carson’s fixative, paraformaldehyde and Bouin’s solution; moderate for formol saline and Clarke’s fixative; and optimal for neutral buffered formalin.

### SMLM of FFPE tissue reveals podocyte structures in normal renal tissue

**Figure 2:**
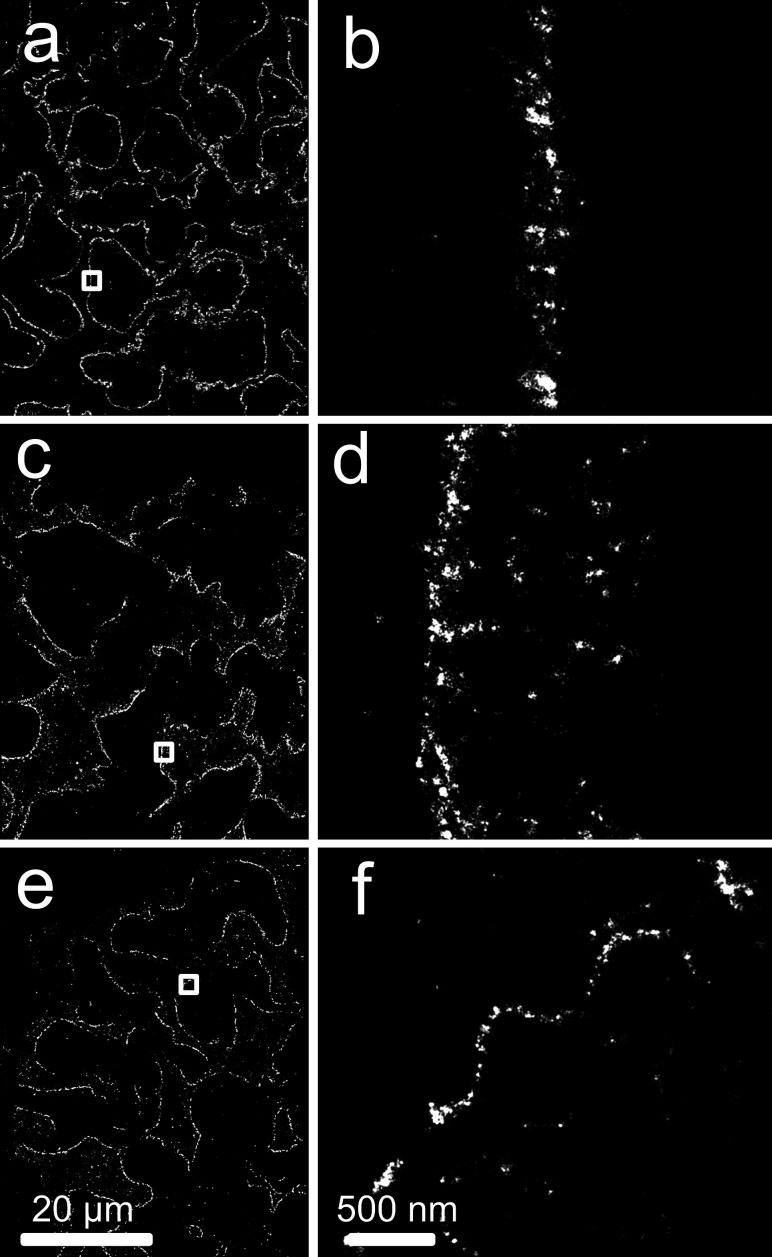
Single-channel SMLM on proteins in healthy renal tissue. (a, b) Laminin, (c, d) podocin and (e, f) nephrin. Boxed regions of (**a, c, e**) shown in (**b, d, f**). SMLM, single-molecule localisation microscopy.

**Figure 3:**
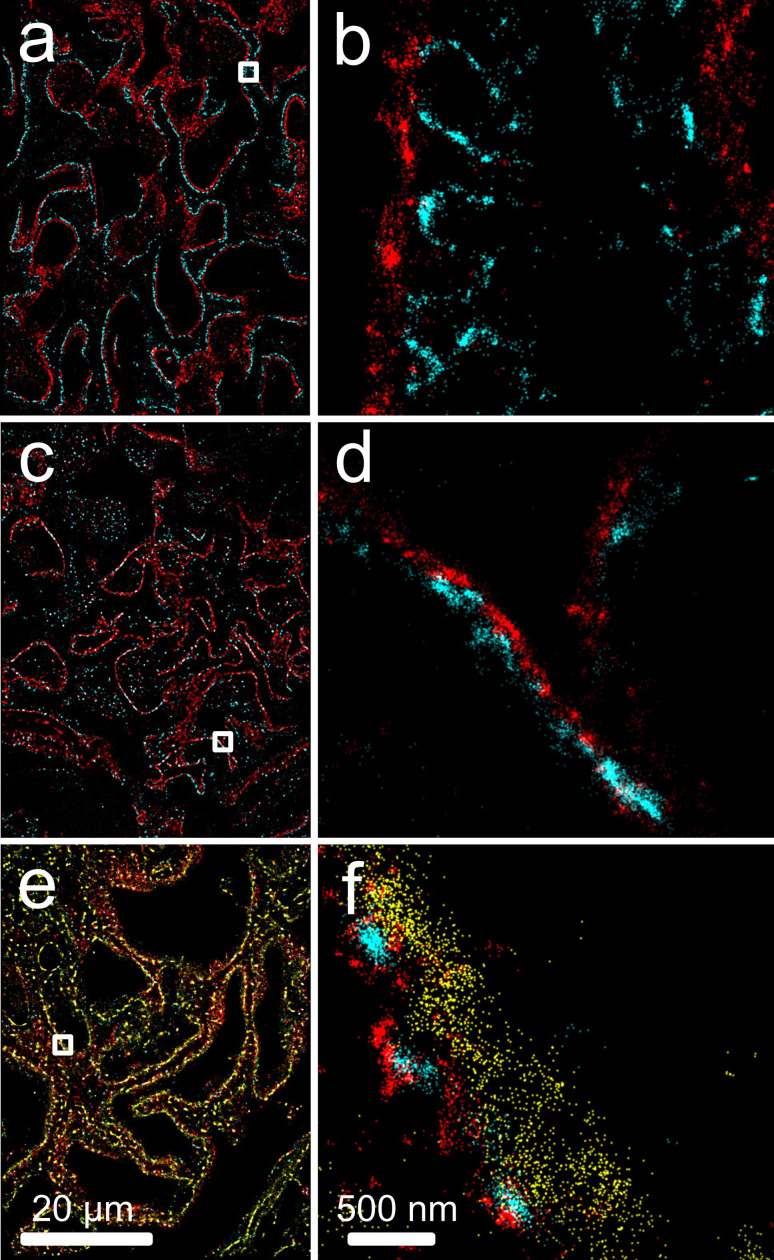
Two-channel and three-channel SMLM on healthy renal tissue. (a, b) Nephrin (CF568, blue) with basement membrane (collagen and laminin, AF647, red); (c, d) laminin (CF568, blue) with basement membrane (collagen and laminin, AF647, red); (e,f) podocin (AF647, red), nephrin (CF568, blue), basement membrane (collagen and laminin, AF488, yellow). Boxed regions of (**a, c, e**) shown in (**b, d, f**). SMLM, single-molecule localisation microscopy.

The optimised super-resolution imaging protocol was established in normal renal FFPE tissue (figure 2a–f). We obtained images with high signal-to-noise ratio, with no significant fixation artefacts, autofluorescence or light scattering for single antibodies staining including against key diagnostic proteins like podocin, nephrin, collagen, laminin, synaptopodin, CD31 (examples shown in figure 2a–f) ; double staining including basement membrane with either nephrin, podocin or collagen IV and further triple staining for basement membrane in conjunction with nephrin and podocin (figure 3a–f). These images were reviewed and discussed with a pathologist (DA) who highlighted important morphological areas for imaging.

### SMLM of FFPE tissue reveals protein patterns in diseased renal tissue

**Figure 4:**
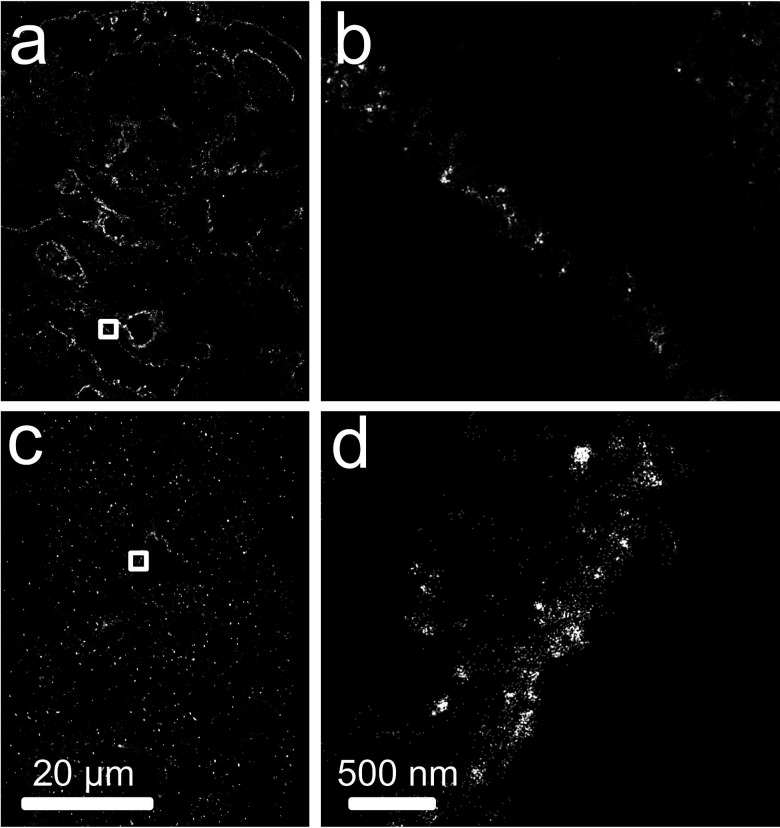
SMLM data on protein expression in immune-mediated diseased including (a, b) IgM in lupus disease and (c, d) IgA in IgA nephropathy. Boxed regions of (a,c) shown in (b, d). SMLM, single-molecule localisation microscopy.

**Figure 5:**
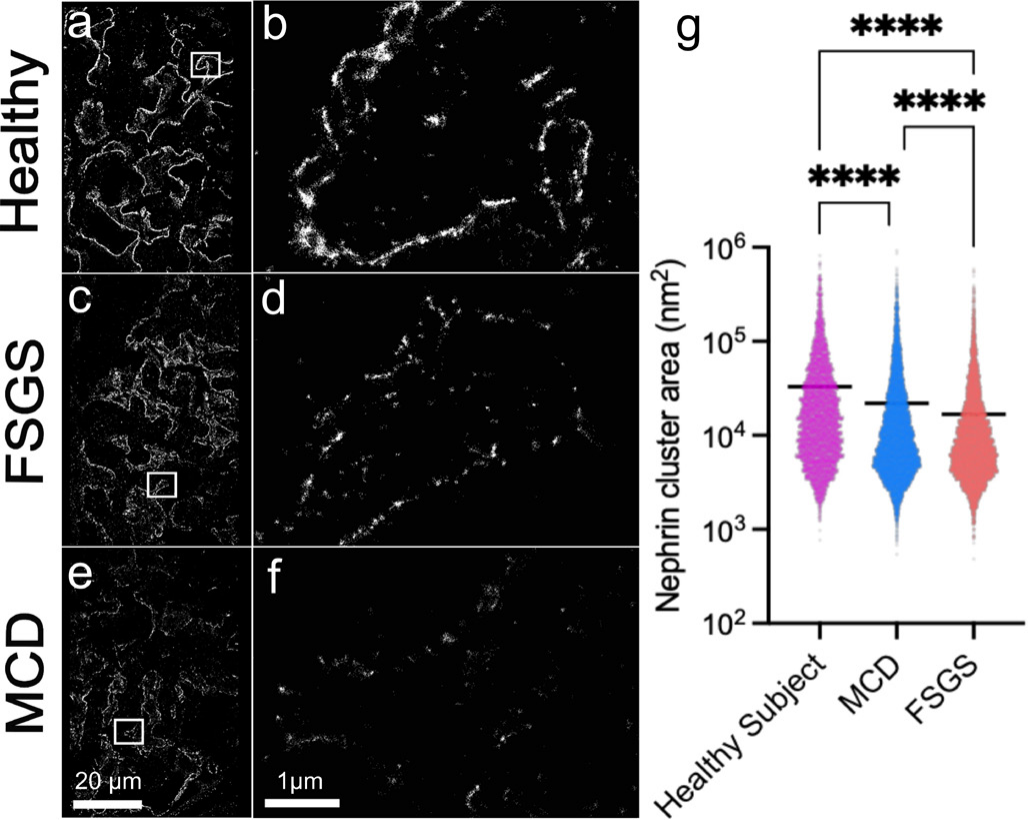
Nephrin distributions (**a–f**) and cluster area comparison (**g**) in healthy (a, b), FSGS (c, d) and MCD (e, f) tissue. Boxed regions of (**a, c, e**) shown in (**b, d, f**); (**g**) ****: p<0.0001 (two-way ANOVA with Tukey’s multiple comparisons test). FSGS, focal segmental glomerulosclerosis; MCD, minimal change disease; ANOVA, Analysis of variance.

We then transferred our protocol into diseased FFPE renal tissue sections including immune meditated diseases (figure 4a–d), MCD and FSGS (figure 5a–g). We again obtained super-resolution images with high signal-to-noise ratio, with no obvious autofluorescence or light scattering against key diagnostic features including in MCD and FSGS for nephrin, in Lupus nephropathy for IgM and in IgA nephropathy for IgA.

### SMLM of FFPE tissue reveals changes in podocyte protein distribution in MCD and FSGS

**Figure 6:**
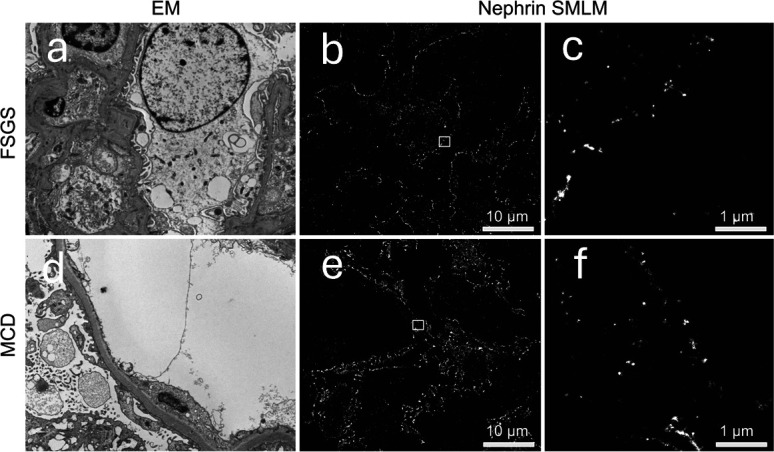
Matched EM and SMLM data FSGS and MCD. (**a**) EM image of FSGS shows patchy distribution of foot process effacement (**b,c**) corresponding single channel SMLM shows disrupted patchy expression of Nephrin protein with focal remaining small Nephrin clusters; boxed region in (b) seen in (c). (**d**) EM image of MCD medical renal biopsy shows extensive foot process effacement, (**e,f**) corresponding single channel SMLM images shows correlating severely disrupted pattern of Nephrin protein expression; boxed area (e) seen in (**f**). EM, electron microscopy; FSGS, focal segmental glomerulosclerosis; MCD, minimal change disease; SMLM, single-molecule localisation microscopy.

We acquired single molecule localisations of nephrin from tissue sections from patients diagnosed as ‘normal’ (without renal disease), with FSGS or with MCD. Two cases for each condition and a minimum of three fields of view (FOVs)/case were used. HDBSCAN is an unsupervised machine learning-based clustering method that was applied on the data to identify the individual nephrin structures. While substantial heterogeneity was observed across cluster morphologies, the distribution of nephrin cluster areas was significantly different between the conditions (p<0.0001, two-way ANOVA with Tukey’s multiple comparison test). Therefore, super-resolution microscopy with a robust fixation method followed by nephrin staining is a promising method for identifying nanoscale structural differences between these renal conditions (figure 5a–g) which is comparable to those changes seen on EM (figure 6a–f).

## Discussion

Super-resolution microscopy on routine FFPE tissue has been reported before but has not yet been adopted into routine clinical services to provide the potential of a rapid and ultrastructural diagnosis.^14^,^17^ The new availability of reliable, easy to use benchtop instruments with good antibodies and robust software changes the paradigm for such an approach.

We were able to establish simple robust protocols for the usage of archival routine clinical medical FFPE renal biopsies to obtain super-resolution microscopy images of medical renal tissue. The oldest material went back over 10 years.

We have demonstrated that the technology works well on routine FFPE tissue, allowing the visualisation of all glomeruli (around 10–25) in the biopsy rather than only the one or two glomeruli which are routinely analysed by gold standard EM. We can demonstrate the key features seen on EM such as podocyte foot processes (podocin), the interpodocyte space (nephrin), the capillary loops (CD31) and basement membrane (collagen and beta laminin 2), which can be resolved by SMLM. Furthermore, we can determine the position, amount and relationships of specific key proteins and their location within the glomeruli.

The technology uses standard methods of tissue preparation and staining that can be widely applied and used on serial sections after the routine diagnostic H&E. This allows a close correlation to light microscopy features as well as special or immunohistochemical stains. This method has a rapid turnaround time of 1–2 days, faster than most centres can obtain which are currently using EM. It does not require a separate clinical or fixative pathway in comparison to EM, so decisions to sample for ultrastructure do not need to be made at the time of fixation and SMLM can be undertaken at any time even years after the original sampling.

Our next steps are to determine the best analysis methods including the use of artificial intelligence techniques. We will then expand the number of normal and diseased cases analysed and move on to other renal pathologies.

Overall, SMLM opens up subcellular microscopy to histopathologists on routinely fixed FFPE tissue. We are currently translating these results into other clinically important disease processes.

## Supplementary material

10.1136/jcp-2024-209853 online supplemental file 1

## Data Availability

All data relevant to the study are included in the article or uploaded as supplementary information.
