# Supplementary material for: Single-molecule localisation microscopy (SMLM) is feasible in human and animal formalin fixed paraffin embedded (FFPE) tissues in medical renal disease
Source: J Clin Pathol. 2025 Jan 13;78(5):e209853. doi: 10.1136/jcp-2024-209853 (PMC12015034; doi:10.1136/jcp-2024-209853)
Supplement: online supplemental file 1 [file jcp-78-5-s001.pdf]

SUPPLEMENTARY MATERIAL

Table 1 and 2: Overview of primary and secondary antibodies, supplier, antigen retrieval method, and solution, concentration and incubation time of selected antibodies used.

Table 1

Primary Antibody	Supplier	Antibody species	Antigen retrieval method	Antigen retrieval solution	Antibody concentra-tion	Incubation conditions
Podocin	Sigma (HPA049486)	Rabbit	HIER using pressure cooker, 125°C for 5mins	Reveal Decloaker 10x (Citrate based)<br>Biocare medical (RV1000M)	1 in 50	Room temperature, 1 hour
CD31	Agilent (M0823)	Mouse	HIER using pressure cooker, 125°C for 5mins	Reveal Decloaker 10x (Citrate based)<br>Biocare medical (RV1000M)	1 in 100	Room temperature, 1 hour
Nephrin	R&D Systems (AF4269)	Sheep	HIER using pressure cooker, 125°C for 5mins	Reveal Decloaker 10x (Citrate based)<br>Biocare medical (RV1000M) solution	1 in 100	Room temperature, 1 hour
Laminin b2	Atlas (aMAb91097)	Mouse	HIER using pressure cooker, 125°C for 5mins	Reveal Decloaker 10x (Citrate based)<br>Biocare medical (RV1000M)	1 in 50	Room temperature, 1 hour
Alpha Actin	ThermoFisher Scientific (MA5-32794)	Rabbit	HIER using pressure cooker, 125°C for 5mins	Reveal Decloaker 10x (Citrate based)<br>Biocare medical (RV1000M)	1in 100	Room temperature, 1 hour
Synaptopodin	R&D systems (918842)	Mouse	HIER using pressure cooker, 125°C for 5mins	Borg Decloaker RTU (EDTA based)<br>Biocare medical (BD1000G1)	1 in 20	Room temperature, 1 hour
Collagen IV	Abcam ab236640	Rabbit	HIER using pressure cooker, 125°C for 5mins	Reveal Decloaker 10x (Citrate based)<br>Biocare medical (RV1000M)	1 in 50	Room temperature, 1 hour
Collagen IV with Alexa Fluor™ 647	ThermoFisher<br>51-9871-80		HIER using pressure cooker, 125°C for 5mins	Reveal Decloaker 10x (Citrate based)<br>Biocare medical (RV1000M)	1 in 50	4°C, overnight

Table 2

**Secondary Antibody**	**Supplier**	**Antibody concentration, diluted in PBS**	**Incubation conditions**
Donkey anti-Rabbit IgG (H+L) Highly Cross-Adsorbed Secondary Antibody, Alexa Fluor™ 647, Invitrogen™	Invitrogen A31573	1 in 500	Room temperature, 1 hour
Donkey anti-Mouse IgG (H+L) Highly Cross-Adsorbed Secondary Antibody, Alexa Fluor™ 647, Invitrogen™	Invitrogen A31571	1 in 500	Room temperature, 1 hour
Donkey anti-Sheep IgG (H+L) Cross-Adsorbed Secondary Antibody, Alexa Fluor™ 647, Invitrogen™	Invitrogen A21448	1 in 500	Room temperature, 1 hour
Goat anti-Mouse IgG (H+L), F(ab')2 fragment, CF568	Biotium 20109-1	1 in 1000	Room temperature, 1 hour
Goat anti-Rabbit IgG (H+L), F(ab')2 fragment, CF568	Biotium 20099-1	1 in 1000	Room temperature, 1 hour
